# Supplementary material for: Post-ER Stress Biogenesis of Golgi Is Governed by Giantin
Source: Cells. 2019 Dec 13;8(12):1631. doi: 10.3390/cells8121631 (PMC6953117; doi:10.3390/cells8121631)
Supplement: Supplementary file 1 [file cells-08-01631-s001.zip › cells-653076-supplementary/supplementary-layout/cells-653076-proof-supplementary-layout.docx]

Article

Post-ER Stress Biogenesis of Golgi Is Governed by Giantin

Cole P. Frisbie ^1^, Alexander Y. Lushnikov ^2^, Alexey V. Krasnoslobodtsev ^2,3^,
Jean-Jack M. Riethoven ^4,5^, Jennifer L. Clarke ^5,6^, Elena I. Stepchenkova ^7,8^ and
Armen Petrosyan ^1,5,9,^*

^1^ Department of Biochemistry and Molecular Biology, University of Nebraska Medical Center, Omaha, NE 68198-5870, USA; [cole.frisbie@unmc.edu](javascript:void(0);)

^2^ Nanoimaging Core Facility, University of Nebraska Medical Center, Omaha, NE 68198-6025, USA; [alushnikov@unmc.edu](javascript:void(0);) (A.Y.L.); [akrasnos@unomaha.edu](javascript:void(0);) (A.V.K.)

^3^ Department of Physics, University of Nebraska-Omaha, Omaha, NE 68182-0266, USA

^4^ Center for Biotechnology, University of Nebraska-Lincoln, Lincoln, NE 68588-0665, USA; [jeanjack@unl.edu](javascript:void(0);)

^5^ The Nebraska Center for Integrated Biomolecular Communication, University of Nebraska-Lincoln, Lincoln, NE 68588-0304, USA; [jclarke3@unl.edu](javascript:void(0);)

^6^ Department of Statistics, University of Nebraska-Lincoln, Lincoln, NE 68583-0963, USA

^7^ Vavilov Institute of General Genetics, Saint-Petersburg Branch, Russian Academy of Sciences, Saint-Petersburg 199034, Russia; [stepchenkova@gmail.com](javascript:void(0);)

^8^ Department of Genetics, Saint-Petersburg State University, Saint-Petersburg 199034, Russia

^9^ The Fred and Pamela Buffett Cancer Center, Omaha, NE 68198-5870, USA

***** Correspondence: apetrosyan@unmc.edu; Tel.: +1402-559-1794; Fax: +1402 559-6650

**Supplemental information**

*
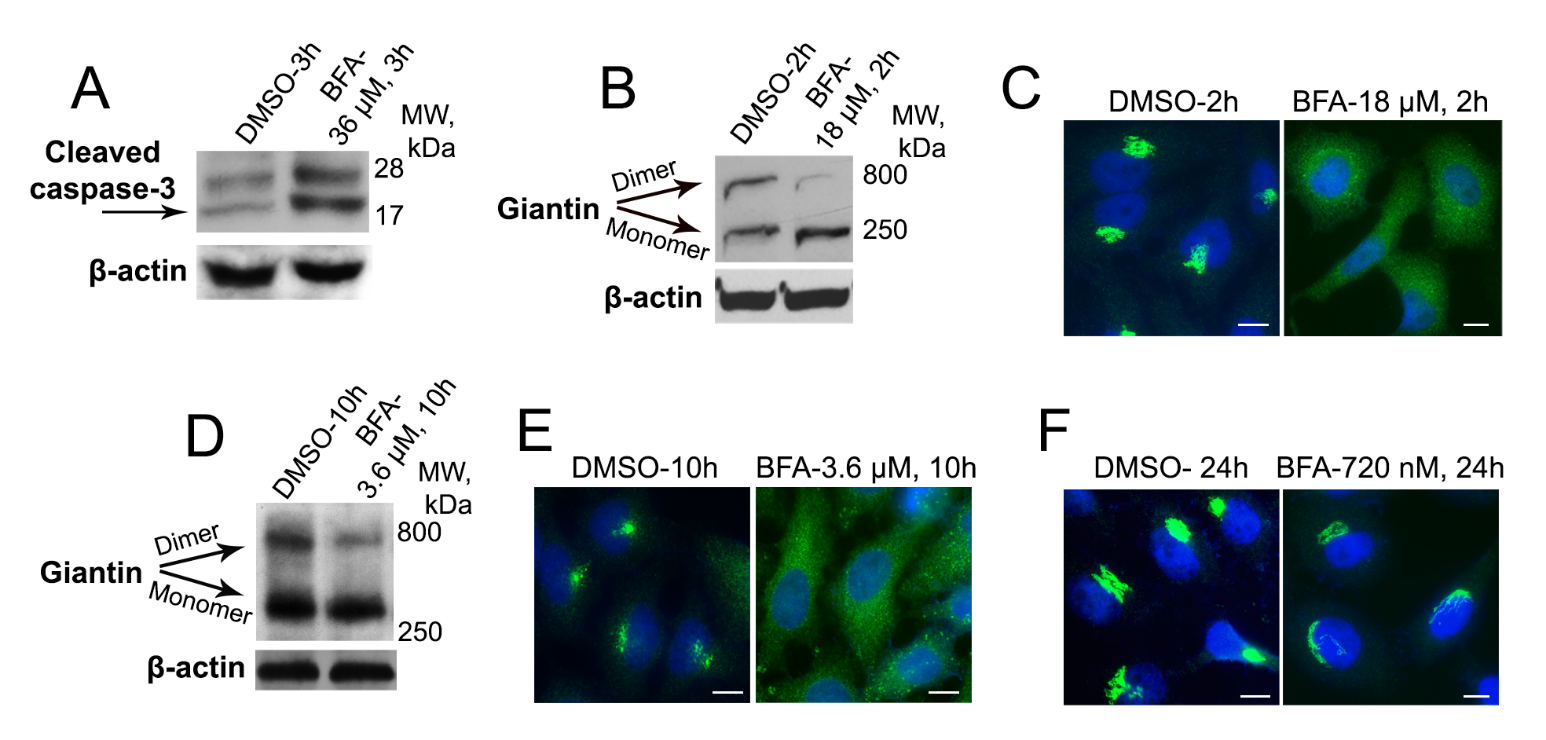
*

**Figure S1**. (**A**) Caspase-3 W-B of the lysates of HeLa cells treated with 36 μM BFA for 3 h or with a corresponding amount of DMSO (control). Samples were prepared under 10% β-mercaptoethanol and run on 15% SDS-PAGE; β-actin was a loading control. The cleaved caspase-3 is indicated by arrow. (**B**) Giantin W-B of the lysates of HeLa cells treated with 18 μM BFA for 2 hr and corresponding amount of DMSO. The lysates were prepared in the presence of 2 mM NEM followed by 5% β-mercaptoethanol and resolved by 4-15% SDS-PAGE. Giantin-dimer and monomer are indicated by arrows. (**C**) Immunostaining of giantin in HeLa cells: control (DMSO-treated) and treated with 18 μM BFA for 2 h. (**D**) Giantin W-B of the lysates of HeLa cells treated with 3.6 μM BFA for 10 h and corresponding amount of DMSO. The lysates were prepared in the presence of 2 mM NEM followed by 5% β-mercaptoethanol and resolved by 4-15% SDS-PAGE. Giantin-dimer and monomer are indicated by arrows. (**E**) Immunostaining of giantin in HeLa cells: control (DMSO-treated) and treated with 3.6 μM BFA for 10 h. (**F**) Immunostaining of giantin in HeLa cells: control (DMSO-treated) and treated with 720 nM BFA for 24 h. Nuclei were counterstained with DAPI (blue). All confocal images were acquired with the same imaging parameters; bars, 10 μm.

*
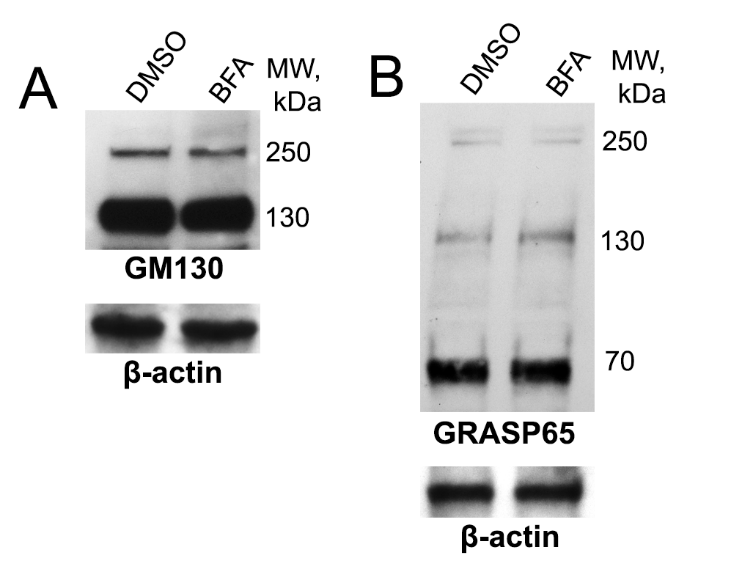
*

**Figure S2**. GM130 (**A**) and GRASP65 (**B**) W-B of the lysates of HeLa cells treated with 36 μM BFA for 1 h or with a corresponding amount of DMSO (control). Samples were prepared under 2 mM NEM followed by 10% β-mercaptoethanol and run on 10% SDS-PAGE; β-actin was a loading control. **A**: The bands around 130 and 250 kDa indicate the monomer and dimer of GM130, accordingly. **B**: The bands around 70, 130 and 250 kDa indicate the monomer, dimer, and oligomers of GRASP65, respectively.


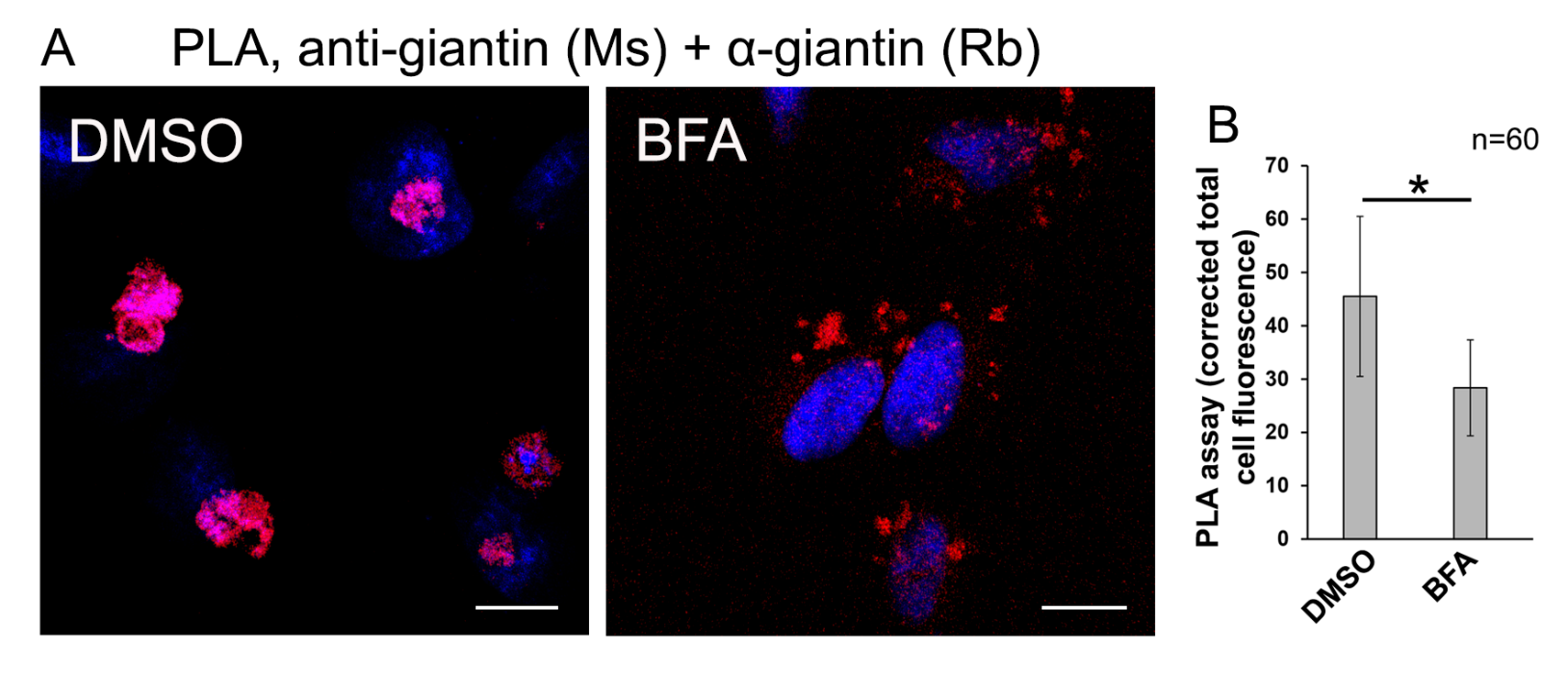


**Figure S3.** (**A**) Proximity ligation assay (PLA) of giantin in HeLa cells (DMSO- and BFA-treated), as analyzed by confocal microscopy; bars, 10 μm. Cells were treated with mouse monoclonal anti-giantin 3-91 aa and rabbit polyclonal anti-giantin 108-157 aa, and then with oligonucleotide-conjugated anti-mouse minus and anti-rabbit plus proximity ligation assay secondary probes. Red punctae indicate PLA signal, nucleus – blue, DAPI; bars, 10 μm. (**B**) Quantitation of proximity ligation is presented as the corrected total fluorescence intensity (a.u.). The results are measured as a mean ± SD; *, p<0.001.


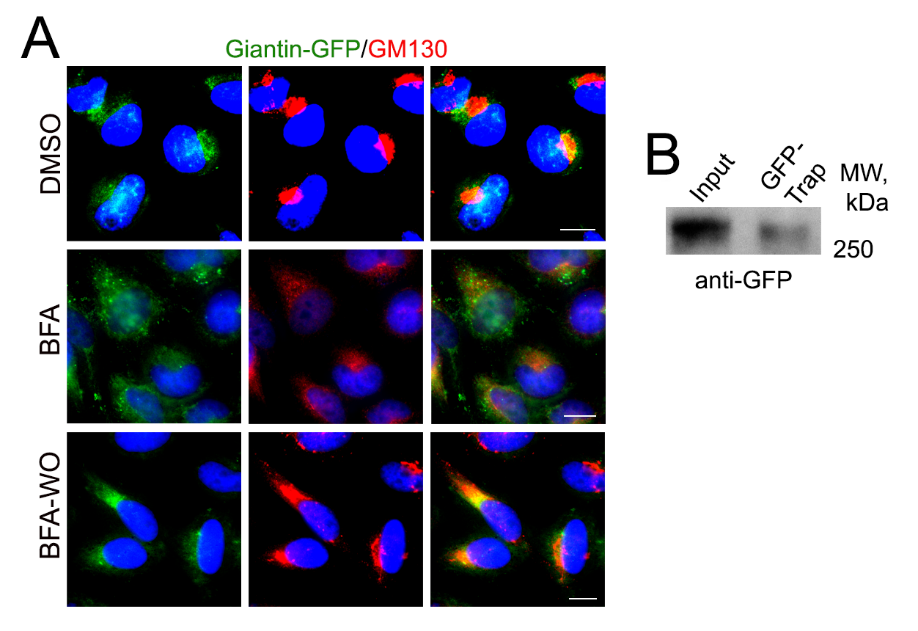


**Figure S4.** (**A**) Confocal immunofluorescence images of Golgi in HeLa cells transfected with giantin-GFP and stained with GM130 (red): control (DMSO-treated), BFA-treated, and BFA-WO for 60 min. The picture was taken by the EVOS M5000 microscope; bars, 10 μm. (**B**) GFP W-B of the lysate of HeLa cells and fraction of GFP-Trap. The samples were prepared under 5% β-mercaptoethanol and resolved by 4-15% SDS-PAGE.

*
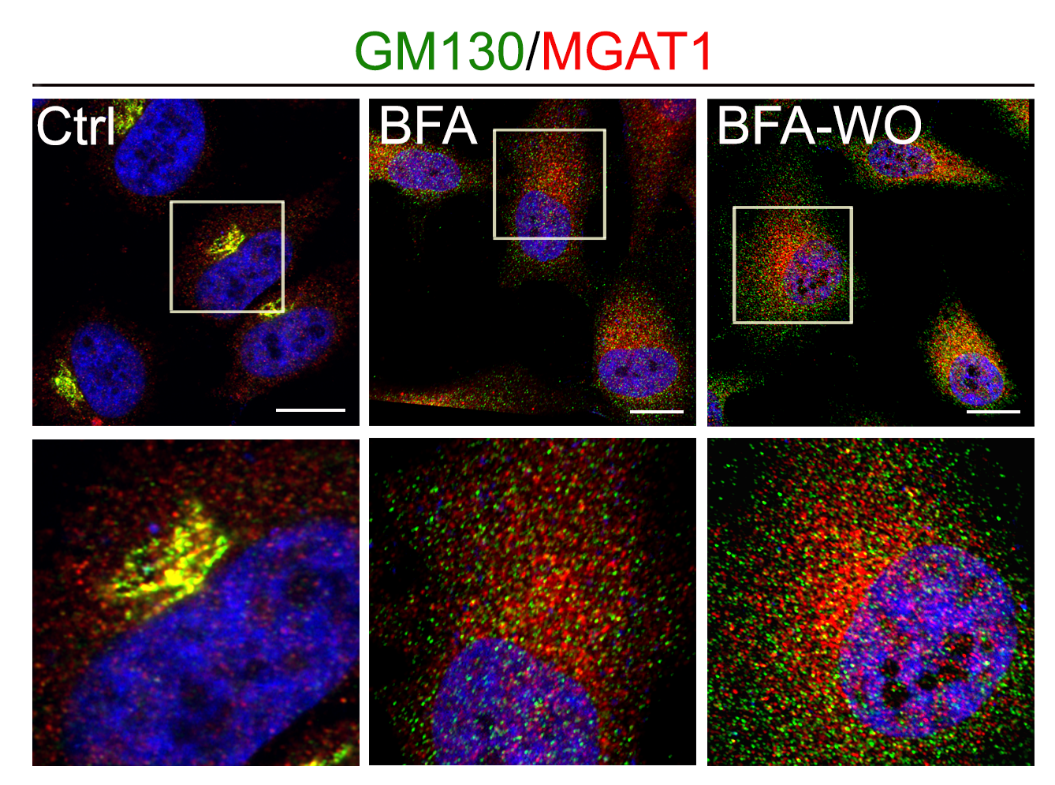
*

**Figure S5. Subcellular distribution of GM130 and MGAT1 in HeLa cells treated with BFA and after BFA washout**. Immunostaining of GM130 and MGAT1 in HeLa cells: control (DMSO-treated), BFA-treated and BFA-WO for 30 min. White boxes indicate Golgi areas enlarged and shown below. Nuclei were counterstained with DAPI (blue). All confocal images were acquired with the same imaging parameters; bars, 10 μm.

*
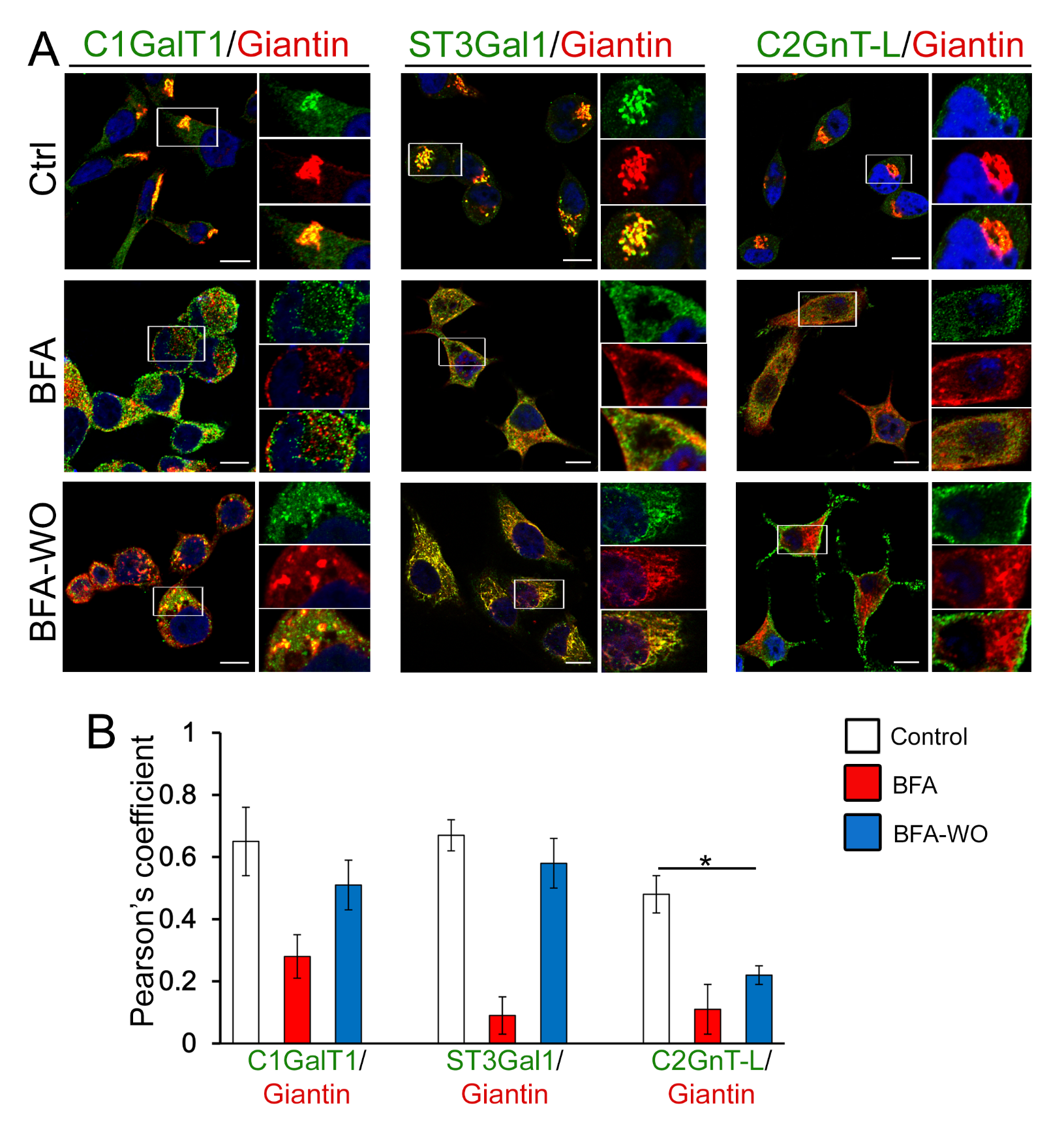
*

**Figure S6. Subcellular distribution of glycosyltransferases and giantin in LNCaP cells treated with BFA and after BFA washout**. (**A**) Confocal immunofluorescence images of C1GalT1, ST3Gal1, C2GnT-L, and giantin in LNCaP (c-28) cells before and after BFA. C1GalT1, ST3Gal1, and C2GnT-L partially colocalize with giantin in cells treated with 36 μM BFA for 60 min. At 30 min BFA WO, C1GalT1 and ST3Gal1 but not C2GnT-L remained colocalized with giantin in the reformed, fragmented Golgi membranes. Images in the white boxes are enlarged and displayed as green, red, and merged colors on the right side of each panel. (**B**) Quantification of Pearson’s overlap coefficients for the indicated proteins in cells from A. Data were obtained from 90 cells in three independent experiments. *, p<0.001 compared to control. All confocal images were acquired with the same imaging parameters; bars, 10 μm.


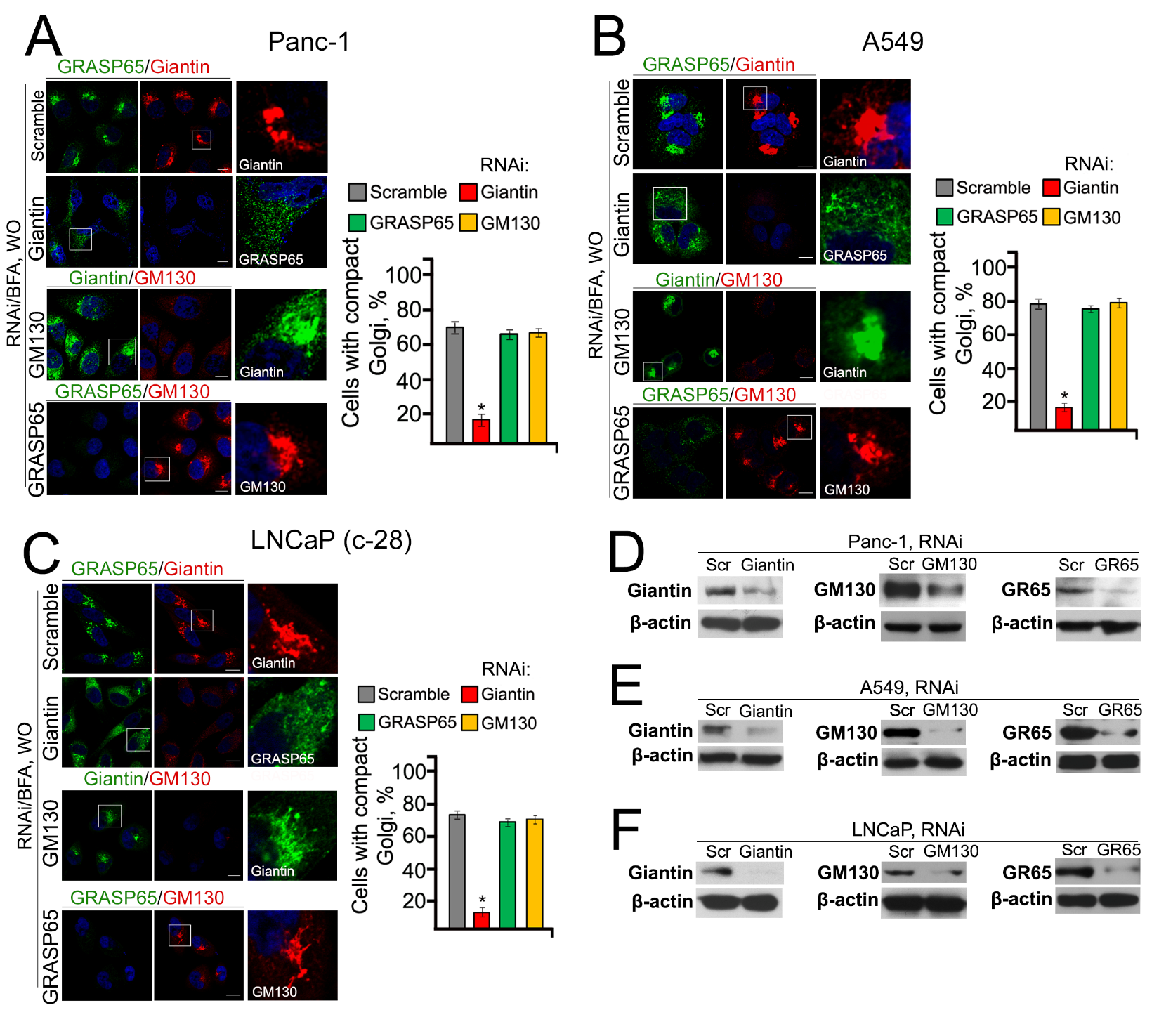


**Figure S7. Giantin is necessary for the restoration of compact Golgi upon BFA washout in Panc-1 (A), A549 (B), and LNCaP (C) cells.** Confocal immunofluorescence images of the Golgi were collected in cells pretreated with scramble, giantin, GM130 or GRASP65 siRNAs followed by exposure to 36 μM BFA for 60 min and then washout for 30 min. Cells lacking detectable giantin, but not GM130 or GRASP65, were unable to re-form compact Golgi. Images in the white box are enlarged and displayed as either green or red channel on the right side. All confocal images were acquired with the same imaging parameters; bars, 10 μm. Nuclei were counterstained with DAPI (blue). Quantification of cells with compact Golgi as shown in A, B, and C, respectively. Data were obtained from 90 cells in three independent experiments. *, p<0.001. (**D-F**) Giantin, GM130, and GRASP65 W-B of lysates of Panc-1 (D), A549 (E), and LNCaP (F) cells treated with the corresponding siRNAs; β-actin was a loading control.


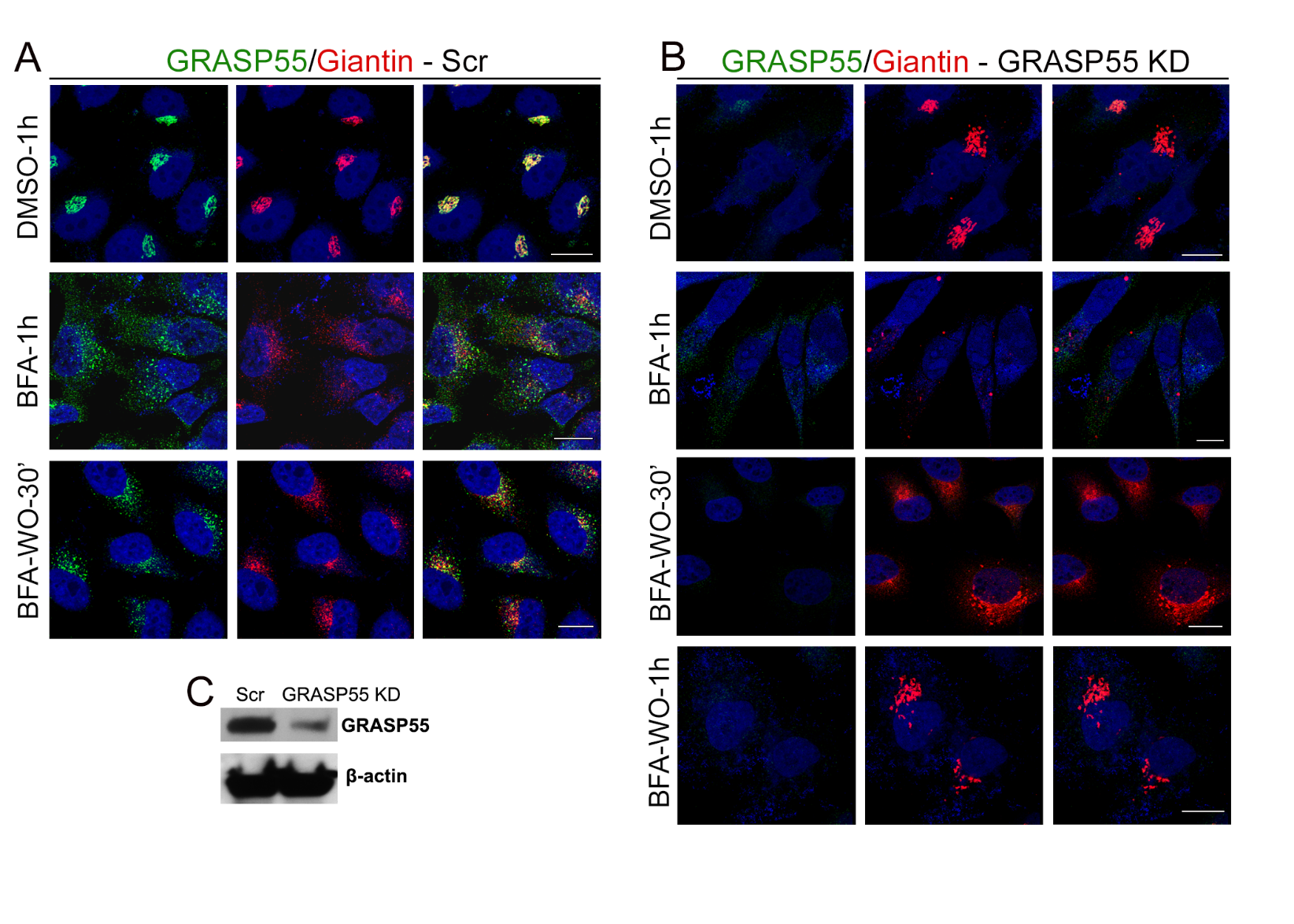


**Figure S8. GRASP55 is not required for the restoration of compact Golgi upon BFA washout in HeLa cells.** (**A**) Confocal immunofluorescence images of GRASP55 (green) and giantin (red) were collected in cells pretreated with scramble siRNAs for 72 h followed by exposure to 36 μM BFA for 60 min and then washout for 30 min. Control cells were treated with the corresponding amount of DMSO. (**B**) Confocal immunofluorescence images of GRASP55 (green) and giantin (red) were collected in cells pretreated with GRASP55 siRNAs for 72 h followed by exposure to 36 μM BFA for 60 min and then washout for 30 and 60 min. All confocal images were acquired with the same imaging parameters; bars, 10 μm. Nuclei were counterstained with DAPI (blue). (**C**) GRASP55 W-B of lysates of HeLa cells treated with scramble and GRASP55 siRNAs; β-actin was a loading control.


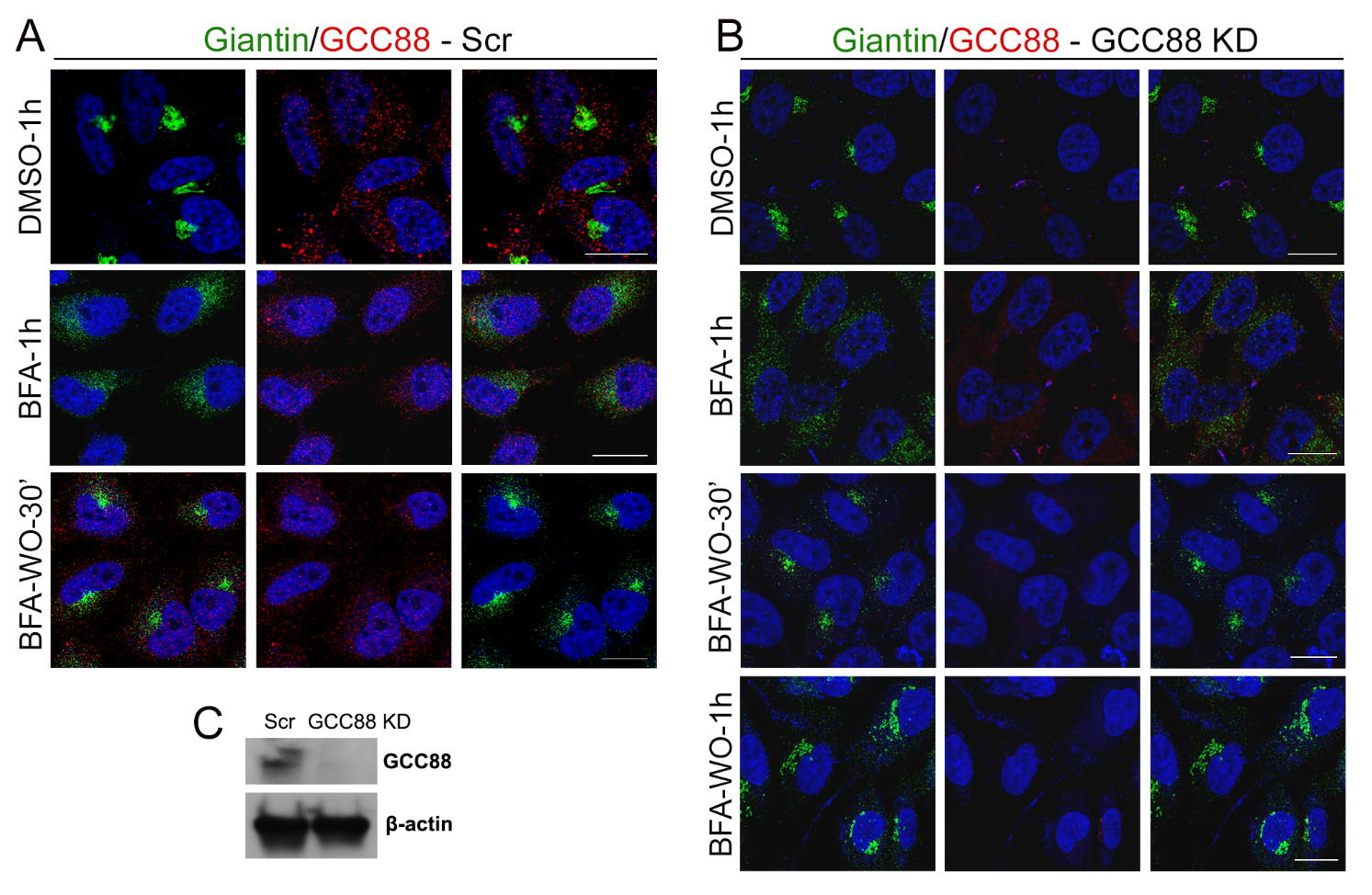


**Figure S9. GCC88 is not required for the restoration of compact Golgi upon BFA washout in HeLa cells.** (**A**) Confocal immunofluorescence images of giantin (green) and GCC88 (red) were collected in cells pretreated with scramble siRNAs for 72 h followed by exposure to 36 μM BFA for 60 min and then washout for 30 min. Control cells were treated with the corresponding amount of DMSO. (**B**) Confocal immunofluorescence images of giantin (green) and GCC88 (red) were collected in cells pretreated with GCC88 siRNAs for 72 h followed by exposure to 36 μM BFA for 60 min and then washout for 30 and 60 min. All confocal images were acquired with the same imaging parameters; bars, 10 μm. Nuclei were counterstained with DAPI (blue). (**C**) GCC88 W-B of lysates of HeLa cells treated with scramble and GCC88 siRNAs; β-actin was a loading control.


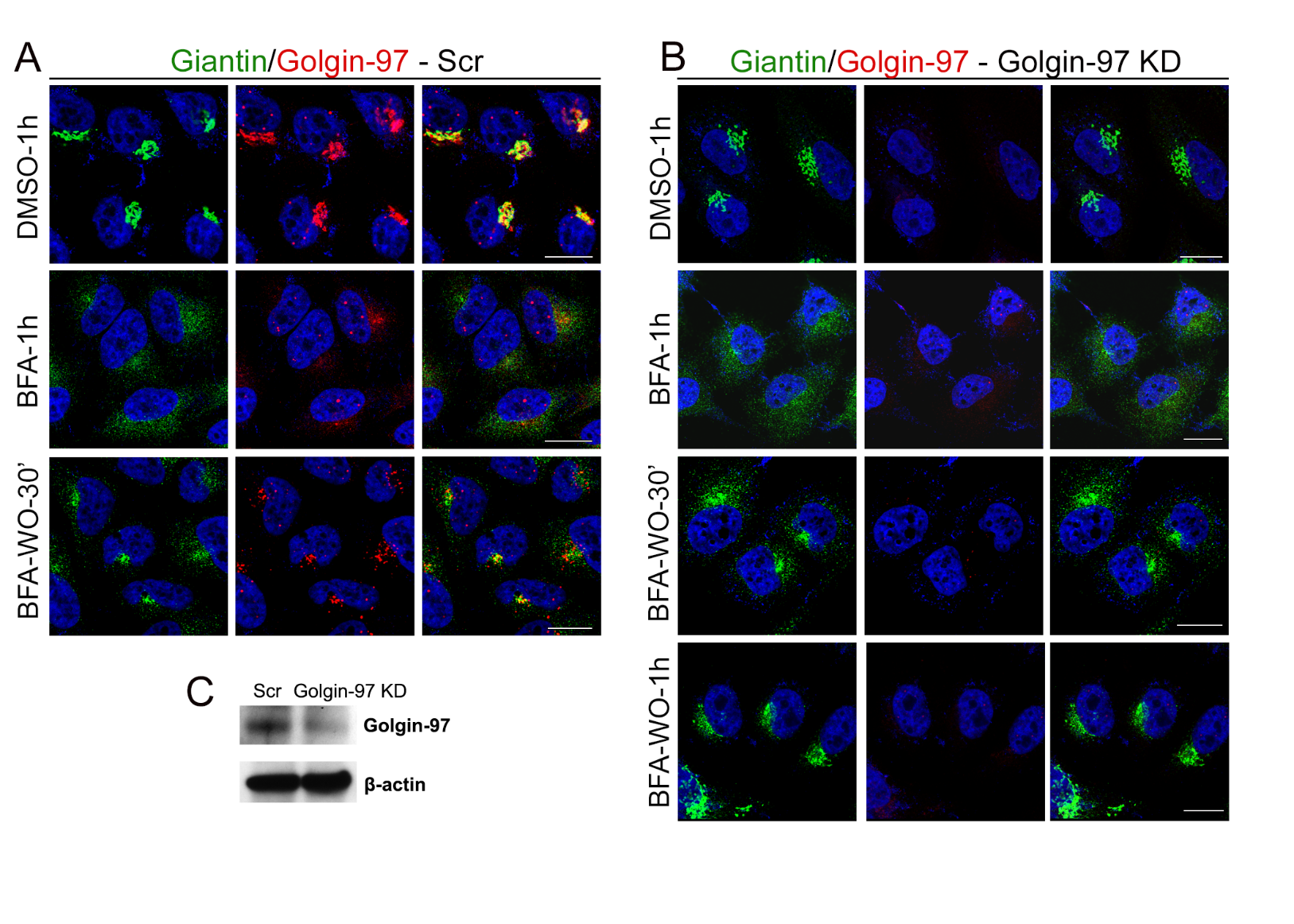


**Figure S10. Golgin-97 is not required for the restoration of compact Golgi upon BFA washout in HeLa cells.** (**A**) Confocal immunofluorescence images of giantin (green) and Golgin-97 (red) were collected in cells pretreated with scramble siRNAs for 72 h followed by exposure to 36 μM BFA for 60 min and then washout for 30 min. Control cells were treated with the corresponding amount of DMSO. (**B**) Confocal immunofluorescence images of giantin (green) and Golgin-97 (red) were collected in cells pretreated with Golgin-97 siRNAs for 72 h followed by exposure to 36 μM BFA for 60 min and then washout for 30 and 60 min. All confocal images were acquired with the same imaging parameters; bars, 10 μm. Nuclei were counterstained with DAPI (blue). (**C**) Golgin-97 W-B of lysates of HeLa cells treated with scramble and Golgin-97 siRNAs; β-actin was a loading control.


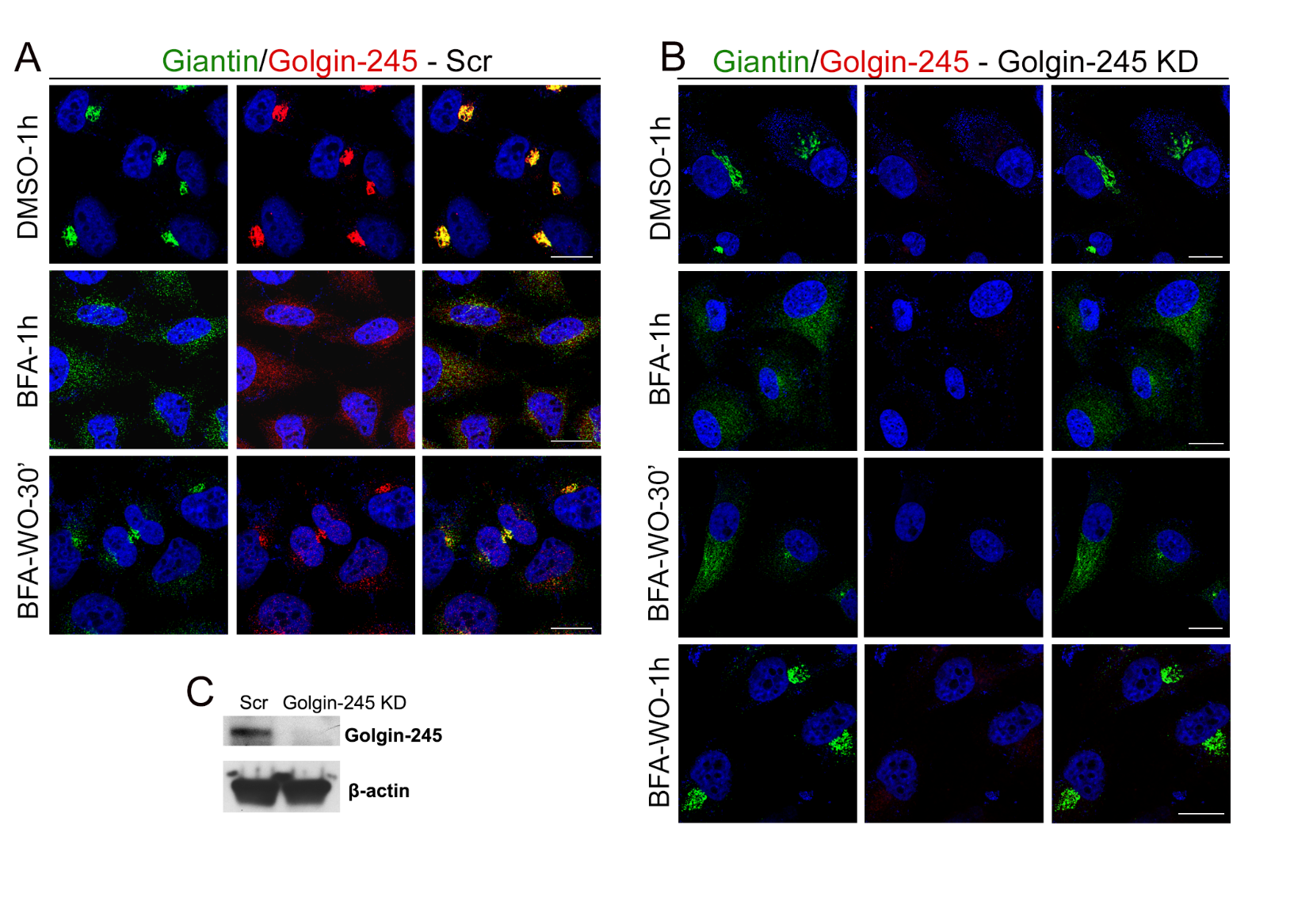


**Figure S11. Golgin-245 is not required for the restoration of compact Golgi upon BFA washout in HeLa cells.** (**A**) Confocal immunofluorescence images of giantin (green) and Golgin-245 (red) were collected in cells pretreated with scramble siRNAs for 72 h followed by exposure to 36 μM BFA for 60 min and then washout for 30 min. Control cells were treated with the corresponding amount of DMSO. (**B**) Confocal immunofluorescence images of giantin (green) and Golgin-245 (red) were collected in cells pretreated with Golgin-245 siRNAs for 72 h followed by exposure to 36 μM BFA for 60 min and then washout for 30 and 60 min. All confocal images were acquired with the same imaging parameters; bars, 10 μm. Nuclei were counterstained with DAPI (blue). (**C**) Golgin-245 W-B of lysates of HeLa cells treated with scramble and Golgin-245 siRNAs; β-actin was a loading control.


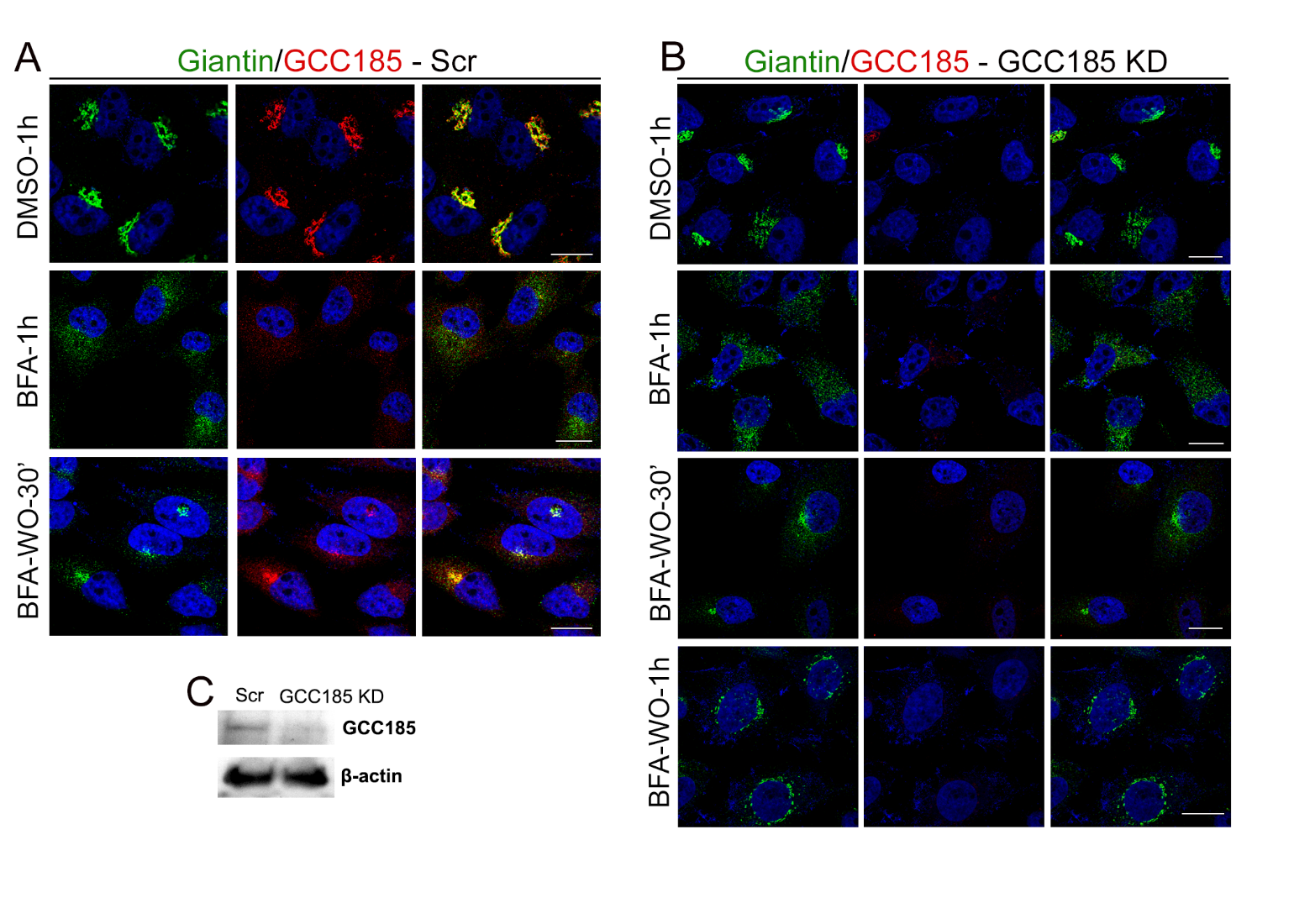


**Figure S12. GCC185 is not required for the restoration of compact Golgi upon BFA washout in HeLa cells.** (**A**) Confocal immunofluorescence images of giantin (green) and GCC185 (red) were collected in cells pretreated with scramble siRNAs for 72 h followed by exposure to 36 μM BFA for 60 min and then washout for 30 min. Control cells were treated with the corresponding amount of DMSO. (**B**) Confocal immunofluorescence images of giantin (green) and GCC185 (red) were collected in cells pretreated with GCC185 siRNAs for 72 h followed by exposure to 36 μM BFA for 60 min and then washout for 30 and 60 min. All confocal images were acquired with the same imaging parameters; bars, 10 μm. Nuclei were counterstained with DAPI (blue). (**C**) GCC185 W-B of lysates of HeLa cells treated with scramble and GCC185 siRNAs; β-actin was a loading control.


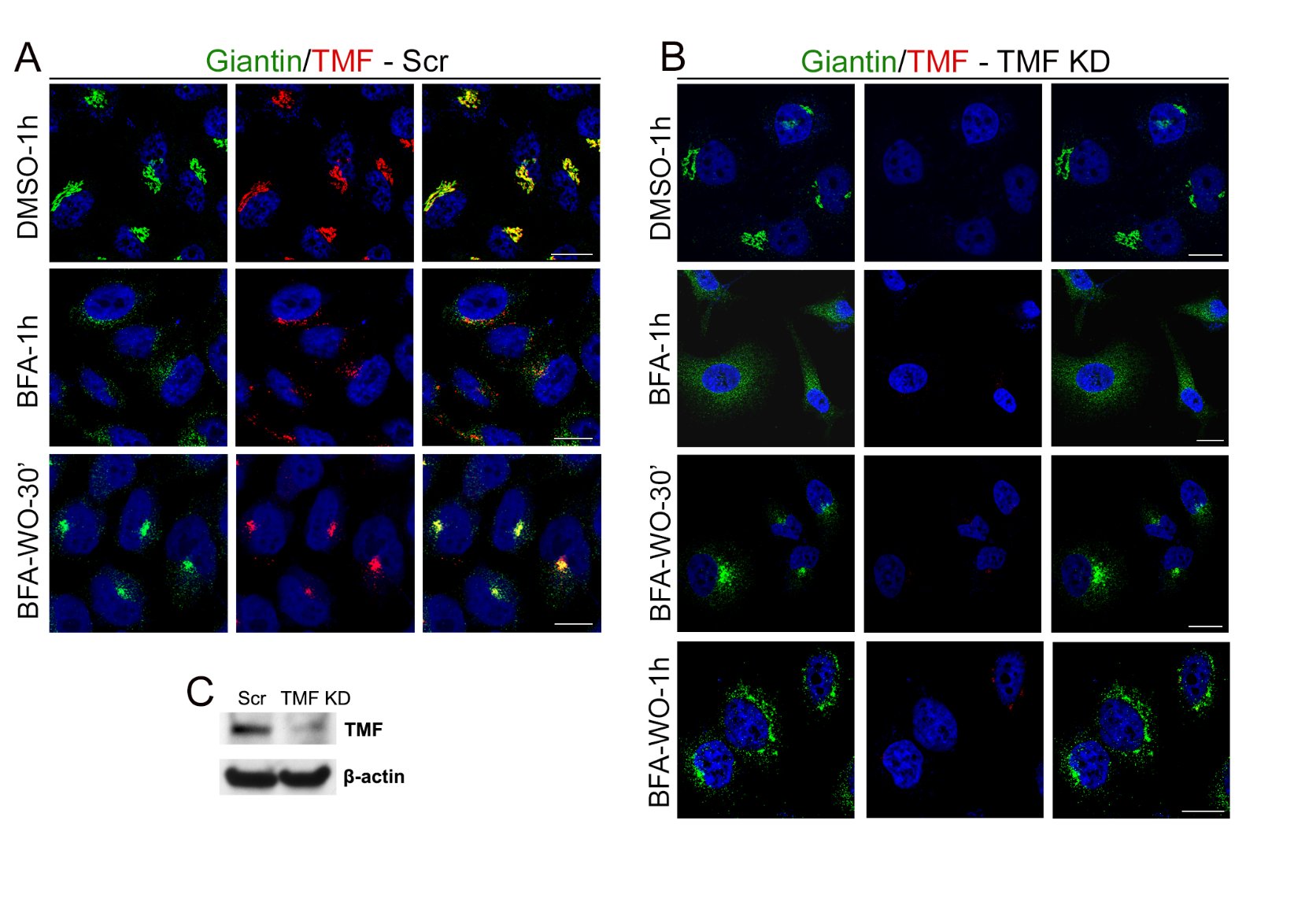


**Figure S13. TMF is not required for the restoration of compact Golgi upon BFA washout in HeLa cells.** (**A**) Confocal immunofluorescence images of giantin (green) and TMF (red) were collected in cells pretreated with scramble siRNAs for 72 h followed by exposure to 36 μM BFA for 60 min and then washout for 30 min. Control cells were treated with the corresponding amount of DMSO. (**B**) Confocal immunofluorescence images of giantin (green) and TMF (red) were collected in cells pretreated with TMF siRNAs for 72 h followed by exposure to 36 μM BFA for 60 min and then washout for 30 and 60 min. All confocal images were acquired with the same imaging parameters; bars, 10 μm. Nuclei were counterstained with DAPI (blue). (**C**) TMF W-B of lysates of HeLa cells treated with scramble and TMF siRNAs; β-actin was a loading control.


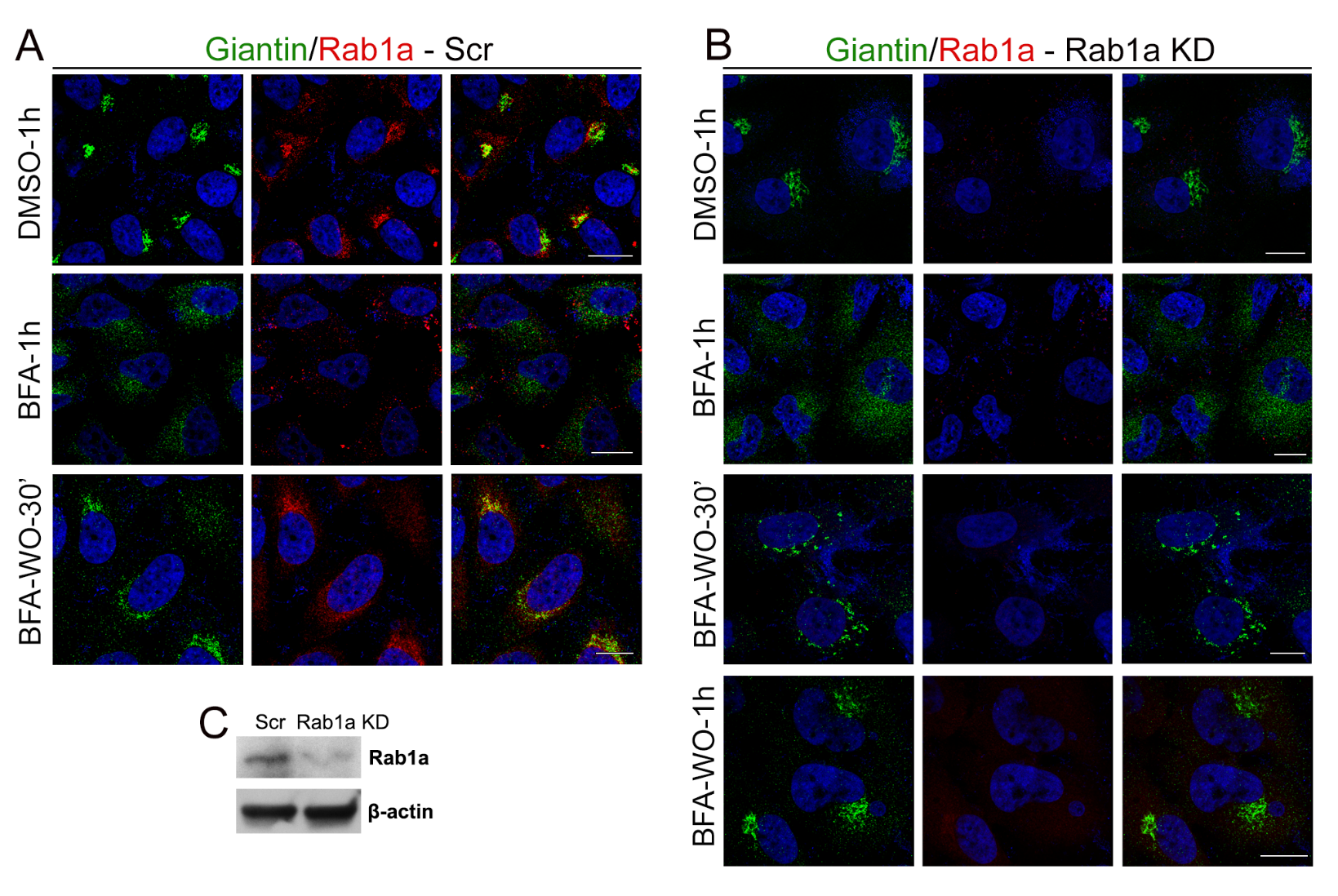


**Figure S14. Rab1a is not required for the restoration of compact Golgi upon BFA washout in HeLa cells.** (**A**) Confocal immunofluorescence images of giantin (green) and Rab1a (red) were collected in cells pretreated with scramble siRNAs for 72 h followed by exposure to 36 μM BFA for 60 min and then washout for 30 min. Control cells were treated with the corresponding amount of DMSO. (**B**) Confocal immunofluorescence images of giantin (green) and Rab1a (red) were collected in cells pretreated with Rab1a siRNAs for 72 h followed by exposure to 36 μM BFA for 60 min and then washout for 30 and 60 min. All confocal images were acquired with the same imaging parameters; bars, 10 μm. Nuclei were counterstained with DAPI (blue). (**C**) Rab1a W-B of lysates of HeLa cells treated with scramble and Rab1a siRNAs; β-actin was a loading control.


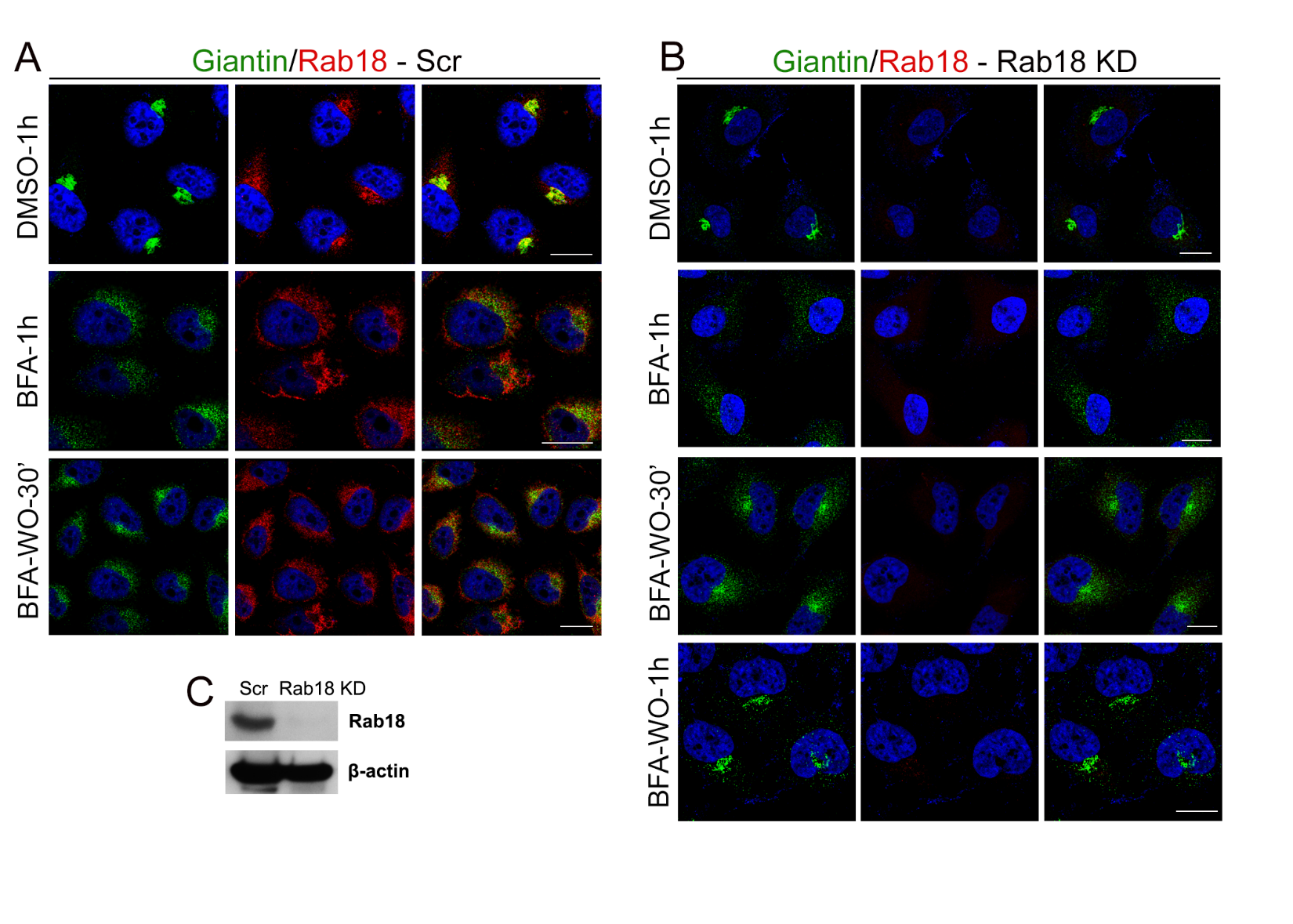


**Figure S15. Rab18 is not required for the restoration of compact Golgi upon BFA washout in HeLa cells.** (**A**) Confocal immunofluorescence images of giantin (green) and Rab18 (red) were collected in cells pretreated with scramble siRNAs for 72 h followed by exposure to 36 μM BFA for 60 min and then washout for 30 min. Control cells were treated with the corresponding amount of DMSO. (**B**) Confocal immunofluorescence images of giantin (green) and Rab18 (red) were collected in cells pretreated with Rab18 siRNAs for 72 h followed by exposure to 36 μM BFA for 60 min and then washout for 30 and 60 min. All confocal images were acquired with the same imaging parameters; bars, 10 μm. Nuclei were counterstained with DAPI (blue). (**C**) Rab18 W-B of lysates of HeLa cells treated with scramble and Rab18 siRNAs; β-actin was a loading control.


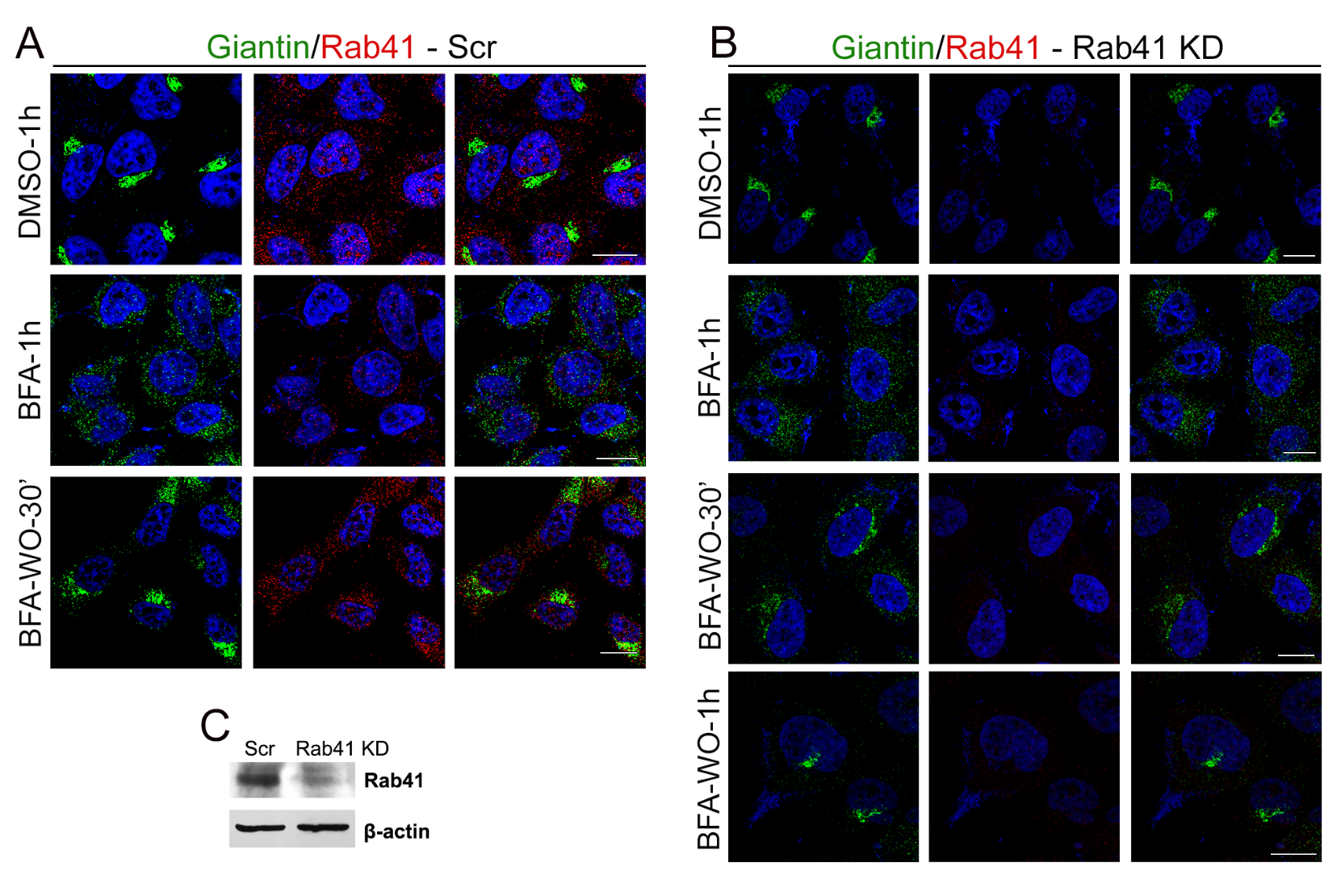


**Figure S16. Rab41 is not required for the restoration of compact Golgi upon BFA washout in HeLa cells.** (**A**) Confocal immunofluorescence images of giantin (green) and Rab41 (red) were collected in cells pretreated with scramble siRNAs for 72 h followed by exposure to 36 μM BFA for 60 min and then washout for 30 min. Control cells were treated with the corresponding amount of DMSO. (**B**) Confocal immunofluorescence images of giantin (green) and Rab41 (red) were collected in cells pretreated with Rab41 siRNAs for 72 h followed by exposure to 36 μM BFA for 60 min and then washout for 30 and 60 min. All confocal images were acquired with the same imaging parameters; bars, 10 μm. Nuclei were counterstained with DAPI (blue). (**C**) Rab41 W-B of lysates of HeLa cells treated with scramble and Rab41 siRNAs; β-actin was a loading control.

**
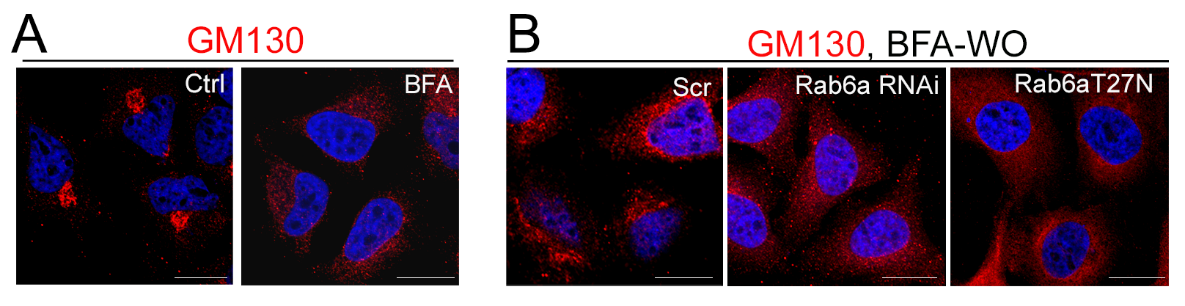
**

**Figure S17.** (**A**) Confocal immunofluorescence images of GM130 in HeLa cells before and after BFA treatment (36 μM BFA for 60 min). (**B**) GM130 immunostaining in HeLa cells after 60 min of BFA-WO and transfected with scramble, Rab6a siRNAs, and dominant-negative (GDP-bound) Rab6a(T27N). All confocal images acquired with the same imaging parameters; bars, 10 μm.

**Figure S18. Kernel density plots of three measures of cisternal changes associated with giantin knockdown (KD).** First column: cisternal length (mcm), ctrl (top) and KD (bottom), (p<0.001); Second column: intercisternal distance (mcm), ctrl (top) and KD (bottom), (p<0.001); Third column: number of intercisternal connections, ctrl (top) and KD (bottom), (p<0.001).

**Description of movies**

**Movie S1.**  **Brefeldin A-mediated apoptosis.** HeLa cells expressing GRASP65-GFP were treated with 36 μM BFA for 120 min, and time-lapse images were collected at 75-sec intervals.

**Movie S2.**  **Brefeldin A-mediated redistribution of Golgi.** HeLa cells expressing GRASP65-GFP were treated with 36 μM BFA for 45 min, and time-lapse images were collected at 75-sec intervals.

**Movie S3.**  **Man-I is able to reach the nascent membranes of post-BFA Golgi.** 3D SIM animation of HeLa cells treated with 36 μM BFA for 60 min and then washout for 30 min. Cells were co-stained for GM130 (green) and Man-I (red).

**Movie S4.**  **MGAT1 is unable to reach the nascent membranes of post-BFA Golgi.** 3D SIM animation of HeLa cells treated with 36 μM BFA for 60 min and then washout for 30 min. Cells were co-stained for giantin (green) and MGAT1 (red).

**Movie S5.** Live imaging of HeLa cells co-expressing giantin-GFP and Rab6a-RFP and recovered after BFA for 30 min.
